# A Case of Mistaken Diagnosis

**Published:** 1868-06-15

**Authors:** Wm. L. Coe

**Affiliations:** Morrison, Ill.


### A CASE OF MISTAKEN DIAGNOSIS.

BY DR. WM. L. COE, MORRISON, ILL.

MY friend and neighbor, Dr. N., was called on August 5th, 1867, to see a child about four years of age, and diagnosed—dislocation of the left femur into the thyroid foramen.

The next day Dr. T. saw the child with him, and confirmed the diagnosis, but owing to disagreement as to the treatment to be adopted, there was no persistent attempt made to reduce the dislocation.

August 7, Dr. T. called upon me to assist in the reduction. After consultation with both gentlemen, I consented.

We found the patient in bed, lying upon his back, with the leg and thigh partially flexed, the knee supported by a pillow beneath it. The hip was red, hot, swollen and tender, so that we could not touch it without distressing the little fellow intensely.

We prepared for reduction, and chloroformed the patient. Upon removing the pillow, the leg was readily extended by the side of its fellow, the foot extending an inch and a half below the right one, the leg somewhat adducted, toes inverted, but easily returned to their proper position by a very little force. The left hip was swollen and very hard about the trochanter, gradually diminishing upwards to the crest of the ilium, and downwards to near the middle of the thigh, and somewhat swollen but softer upon the internal part of the thigh and groin.

Upon applying a tape from the anterior superior process of the ilium to the internal malleolus, I found the legs of the same length precisely. On grasping the thigh firmly, and making persistent pressure upon the soft parts, I found the head of the femur wos not in thyroid hole, but *was* in the acetabulum. Of course there was no dislocation.

Now, how to account for the position of the feet.

Upon further examination, we found the pelvis presented the same obliquity, the left ilium carried downwards, and the transverse axis of the pelvis at an obtuse angle, with a line drawn through the body perpendicularly. We then turned the boy upon the face, and on tracing the spinous processes of the vertebra, found a curvature of the spine in the lumbar region, with the convexity to the left side, which explained the obliquity of the pelvis and consequent extension of the left leg and foot.

From the mother we learned that about ten days before, she had taken the child to a friend's, and while there he had received a slight injury, causing him to cry a little, but soon recovered and returned to play. The child said; "I went to *Danma's* and fell off the *teeter*." In a day or two after, he complained of his hip hurting him, and walked with some difficulty, which all constantly increased, with swelling and tenderness, until the day before Dr. N. saw him, when he became confined to the bed.

Here was the solution — a slight bruise on the hip produced inflammation of the parts, the tissues all partook of the increased action, muscular contraction became painful and was abandoned, either by an effort of the will or involuntarily, the muscles of the spine sympathized, and all became *practi-*

*cally paralyzed*, allowing the muscles of the right side, retaining their normal contractility, to draw the right hip upwards, curving the spine, and producing the appearances described, and well simulating the thyroid dislocation.

In a few days after this, suppuration took place, an abscess was formed in the hip just about the trochanter major, a lancet was passed in, the pus evacuated, and the boy recovered perfectly, with the feet parallel and on the same plane, the pelvis and all the parts being restored to their normal position.

I should not trouble your readers with this small matter, but that a statement of failures and mistakes often serves to fix principles upon the mind more firmly than successes, and I wish to put young members of the profession on their guard in diagnosis.

My practical application is, *Make thorough investigation in all cases, do not jump at conclusions*, and thus avoid the chagrin of confessing to the commission of blunders.

